# The Gonococcal Transcriptome during Infection of the Lower Genital Tract in Women

**DOI:** 10.1371/journal.pone.0133982

**Published:** 2015-08-05

**Authors:** Ryan McClure, Kathleen Nudel, Paola Massari, Brian Tjaden, Xiaohong Su, Peter A. Rice, Caroline A. Genco

**Affiliations:** 1 Department of Medicine, Section of Infectious Diseases, Boston University School of Medicine, Boston, MA, United States of America; 2 Department of Microbiology, Boston University School of Medicine, Boston, MA, United States of America; 3 Department of Computer Science, Wellesley College, Wellesley, MA, United States of America; 4 STD Clinic, Institute of Dermatology, Chinese Academy of Medical Sciences & Peking Union Medical College, Nanjing 210042, China; 5 Department of Medicine, Division of Infectious Diseases and Immunology, University of Massachusetts Medical School, Worcester, MA, United States of America; University of Würzburg, GERMANY

## Abstract

Gonorrhea is a highly prevalent disease resulting in significant morbidity worldwide, with an estimated 10^6^ cases reported annually. *Neisseria gonorrhoeae*, the causative agent of gonorrhea, colonizes and infects the human genital tract and often evades host immune mechanisms until successful antibiotic treatment is used. The alarming increase in antibiotic-resistant strains of *N*. *gonorrhoeae*, the often asymptomatic nature of this disease in women and the lack of a vaccine directed at crucial virulence determinants have prompted us to perform transcriptome analysis to understand gonococcal gene expression patterns during natural infection. We sequenced RNA extracted from cervico-vaginal lavage samples collected from women recently exposed to infected male partners and determined the complete *N*. *gonorrhoeae* transcriptome during infection of the lower genital tract in women. On average, 3.19% of total RNA isolated from female samples aligned to the *N*. *gonorrhoeae* NCCP11945 genome and 1750 gonococcal ORFs (65% of all protein-coding genes) were transcribed. High expression *in vivo* was observed in genes encoding antimicrobial efflux pumps, iron response, phage production, pilin structure, outer membrane structures and hypothetical proteins. A parallel analysis was performed using the same strains grown *in vitro* in a chemically defined media (CDM). A total of 140 genes were increased in expression during natural infection compared to growth in CDM, and 165 genes were decreased in expression. Large differences were found in gene expression profiles under each condition, particularly with genes involved in DNA and RNA processing, iron, transposase, pilin and lipoproteins. We specifically interrogated genes encoding DNA binding regulators and iron-scavenging proteins, and identified increased expression of several iron-regulated genes, including *tbpAB* and *fbpAB*, during infection in women as compared to growth *in vitro*, suggesting that during infection of the genital tract in women, the gonococcus is exposed to an iron deplete environment. Collectively, we demonstrate that a large portion of the gonococcal genome is expressed and regulated during mucosal infection including genes involved in regulatory functions and iron scavenging.

## Introduction

The sexually transmitted infection (STI) gonorrhea, caused by the Gram-negative bacterium *Neisseria gonorrhoeae*, is the second most common reportable disease in the U.S. In 2014, there were 331,000 reported cases and the total annual number is estimated to be 800,000 due to underreporting (Centers for Disease Control and Prevention). Gonorrhea is also prevalent worldwide with an estimated 106 million cases annually [1]. The relatively straightforward therapeutic approaches to treat gonococcal infections have been complicated recently by the emergence of antibiotic resistance [2,3]. In 2013, the CDC reported that antibiotic-resistant *N*. *gonorrhoeae* was a major cause for concern in the US and abroad, and a recent study has described cephalosporin-resistant *N*. *gonorrhoeae* in the United States [4].

Like many other human pathogens, *N*. *gonorrhoeae* must adapt to the environment encountered during infection. Control of gene expression results from a number of distinct mechanisms, including DNA binding proteins and regulatory sRNAs that bind to target mRNAs to control their translation and stability [5–8]. DNA binding proteins that are important in infection include sigma factors, such as RpoH, shown to increase transcription of genes involved in stress response and adherence to epithelial cells [9,10], and OxyR, which regulates expression of genes such as catalase, involved in resistance to bactericidal reactive oxygen species [11]. Another well-known factor crucial for a successful host infection is Fur, which regulates intracellular iron levels [12,13]. Targets of Fur include the ferric-binding protein (FbpA), transferrin binding proteins (TbpAB) and lactoferrin binding proteins (LbpAB).

Despite numerous studies that have examined gonococcal gene expression *in vitro*, little is known about how this pathogen regulates gene expression during natural infection. In the female genital tract, *N*. *gonorrhoeae* is exposed to unique conditions, such as low pH [14,15], varying oxygen and iron levels [16–19], and the presence of additional microbes and host cells [20]. In this environment, free iron is scarce and is usually complexed to host iron binding proteins, including transferrin and lactoferrin, thus making the female genital tract an iron-deplete environment [17]. However, during menses iron levels can rise and *N*. *gonorrhoeae* responds to these changes via expression of the Fur protein [17]. Previous work from our group has shown that gonococcal genes encoding the *tbpAB* genes and the *fur* gene itself are differentially regulated during *in vivo* infection [16,17]. However, these studies were carried out using a combination of subset microarrays and qRT-PCR and thus were limited in their sensitivity and detection.

Here, we present the first study based on an RNA deep sequencing (RNA-seq) analysis of *N*. *gonorrhoeae* RNA samples derived directly from cervico-vaginal lavage specimens of infected women. The gonococcal strains isolated from these samples were also grown *in vitro* and the resulting transcriptomes were compared to those expressed during natural infection to define infection-specific gene expression profiles. This analysis has identified gonococcal genes that are highly expressed during cervical infection in women and other genes that are differentially regulated during infection compared to growth *in vitro*.

## Results

### Global analysis of gonococcal gene expression during natural mucosal infection in women

Following RNA extraction and sequencing from four cervico-vaginal lavage samples, cDNA reads were aligned to the three fully sequenced strains of *N*. *gonorrhoeae* in the NCBI database: *N*. *gonorrhoeae* FA1090, NCCP11945 and TCDC-NGO8107, using Rockhopper software [21]. Gonococcal RNA isolated from all subjects aligned most closely to the NCCP11945 strain, a strain isolated from a vaginal specimen in 2008 in Korea [22]. On average, 3.19% of the total RNA from each sample aligned to the *N*. *gonorrhoeae* NCCP11945 genome and ~45% aligned to the human genome. To be considered expressed, a gene had to have a reads per kilobase per million reads (RPKM) value of at least 10. Our analysis detected expression of 1750 gonococcal genes, representing ~ 65% of the gonococcal genome. The level of expression of all genes, regardless of RPKM level in each subject and infecting strain, is summarized in S1 Table. High expression of several similar genes was detected in each of the vaginal samples (Table 1). These similarities in gene expression occurred despite temporal differences in the exposure of female subjects to the infected partner(s), menstrual cycle and degree of cervico-vaginal neutrophilia (Table 2). This core set of similar genes included those encoding outer membrane (*omp3*, NGK_0749) housekeeping (elongation factor P, Tu), stress response (*groES*), phage associated (NGK_1145) and hypothetical proteins (NGK_0472, NGK_2043) (Table 1). In addition to ORFs, 75 short non-coding transcripts representing regulatory small RNAs (described in detail below), were also expressed. It is important to note that several of these highly expressed genes showed similar high expression *in vitro* during growth in CDM. Specific analysis of genes showing changes in expression as a result of infection is discussed below.

**Table 1:** Gonococcal genes* that exhibited the highest expression levels during infection of the female genital tract.

Gene	Product	Expression Subject 1***	Expression Subject 2***	Expression Subject 3***	Expression Subject 4***
**NGK_0225	16S rRNA-processing protein RimM	546	596	566	712
NGK_0472	Hypothetical protein	1085	1142	1554	1737
NGK_0749	Outer membrane protein	653	361	372	955
NGK_0861	Elongation factor P	1675	1074	685	1311
NGK_0873	Glutaredoxin	3711	1994	2460	4432
NGK_0946	Putative secreted protein	643	451	375	1284
NGK_1099	DbhA	1301	1091	722	3655
NGK_1145	Putative phage associated protein	1462	1063	1343	1717
NGK_1876	Omp3	2001	1229	1279	3718
NGK_2043	Hypothetical protein	657	387	371	985
NGK_2298	Hypothetical protein	10792	23821	12739	17690
NGK_2415	Elongation factor Tu	2983	1698	1704	3233
NGK_2431	Elongation factor Tu	2903	1341	1262	2407
NGK_2459	Glutaredoxin	3186	1887	730	2060
NGK_2520	Acyl carrier protein	975	2203	1787	4170
NGK_2558	Co-chaperone GroES	1647	2317	609	979
NGK_2621	F0F1 ATP synthase subunit C	1186	567	1424	758

* Cervico-vaginal lavage samples were examined separately and genes that were in the top 50 most highly expressed genes in **all** 4 subjects are indicated. rRNA, tRNA, and ribosomal protein coding genes have been removed.

** Locus Tag is shown according to *N*. *gonorrhoeae*, NCCP11945 genome

*** Expression levels shown as Reads per Kilobase per Million Reads (RPKM).

**Table 2:** Characteristics of female subjects with uncomplicated gonorrhea.

Subject	Age	Days Since Last Menstrual Cycle	Days Since Last sex-contact with Infected Male	Cervical Culture for *N*. *gonorrhoeae (Ng)* *	Other Microbes Present (Method of Detection)	Vaginal Wet Mount	Cervical PMNs**
1	29	27	10	Pos.	None	Normal	++
2	35	26	4	Pos.	*C*. *trachomatis* (PCR) *U*. *urealyticum* (Culture), *M*. *hominis* (Culture)	Clue cells (BV^+^)	++
3	21	16	11	Pos.	None	Clue cells (BV^+^)	+
4	23	8	1	Neg.***	*U*. *urealyticum* (Culture)	Normal	None

* Thayer—Martin media; *Ng* confirmed by colonial morphology, Gram’s stain and oxidase testing

** Number of cervical polymorphonuclear leukocytes (PMNs) indicated: +, 1–4 PMNs/oif (oil immersion field); ++, >10 PMNs/oif under light microscopy at X1000

*** Subject 4 was negative for *N*. *gonorrhoeae* by culture but positive by PCR

^**+**^ (BV) Bacterial vaginosis.

**Fig 1:**
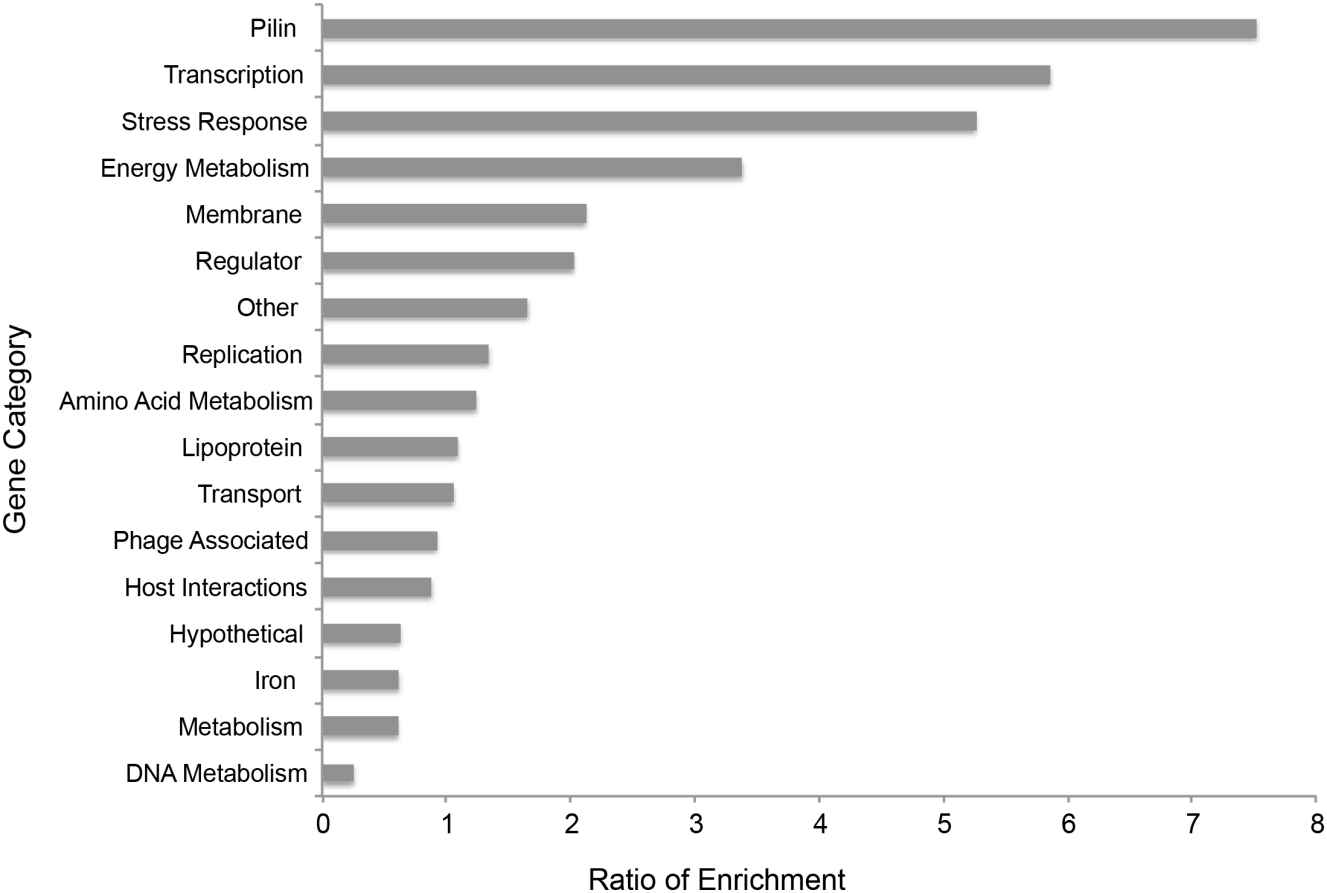
Categorization of top 100 expressed *N*. *gonorrhoeae* genes during natural mucosal infection in women. The top 100 gonococcal genes expressed in 4 infected subjects were categorized based on gene categories using NCBI information from the NCCP11945 strain. Categories are shown on the y-axis with the ratio of enrichment (percentage of genes of a given functional category in the RNA-seq dataset/percentage of genes of that functional category in the whole gonococcal genome) shown on the x-axis. Ribosomal protein, tRNA, and rRNA genes were removed for clarity.

To gain information on general trends of gonococcal responses to infection in women, we categorized and organized the top 100 gonococcal genes that were most highly expressed when RPKM values from all subjects were averaged together. These genes were categorized based on their gene annotation according to strain NCCP11945, which was chosen because sequences aligned most closely to that strain [22]. To determine the ratio of enrichment, these highly expressed genes were first categorized by function. Then, the percentage of genes in each functional category was compared to the percentage of genes within the same functional category in the whole gonococcal genome. The resulting ratio was defined as the “ratio of enrichment”. The most commonly represented enriched categories that were highly expressed included genes associated with pilin, membrane and stress response as well as traditional housekeeping genes such as those involved in transcription, replication and energy metabolism (Fig 1). Expression of these genes showed variable levels in each of the cervico-vaginal samples, but all were expressed at high levels and, as a whole, reflected the profile of the most highly expressed gonococcal genes during natural infection. As above, several of these genes were also highly expressed *in vitro*. A specific analysis of genes that showed changes in expression relative to *in vitro* growth is presented below.

### Expression of *N*. *gonorrhoeae* antimicrobial resistance genes during natural mucosal infection in women

**Fig 2:**
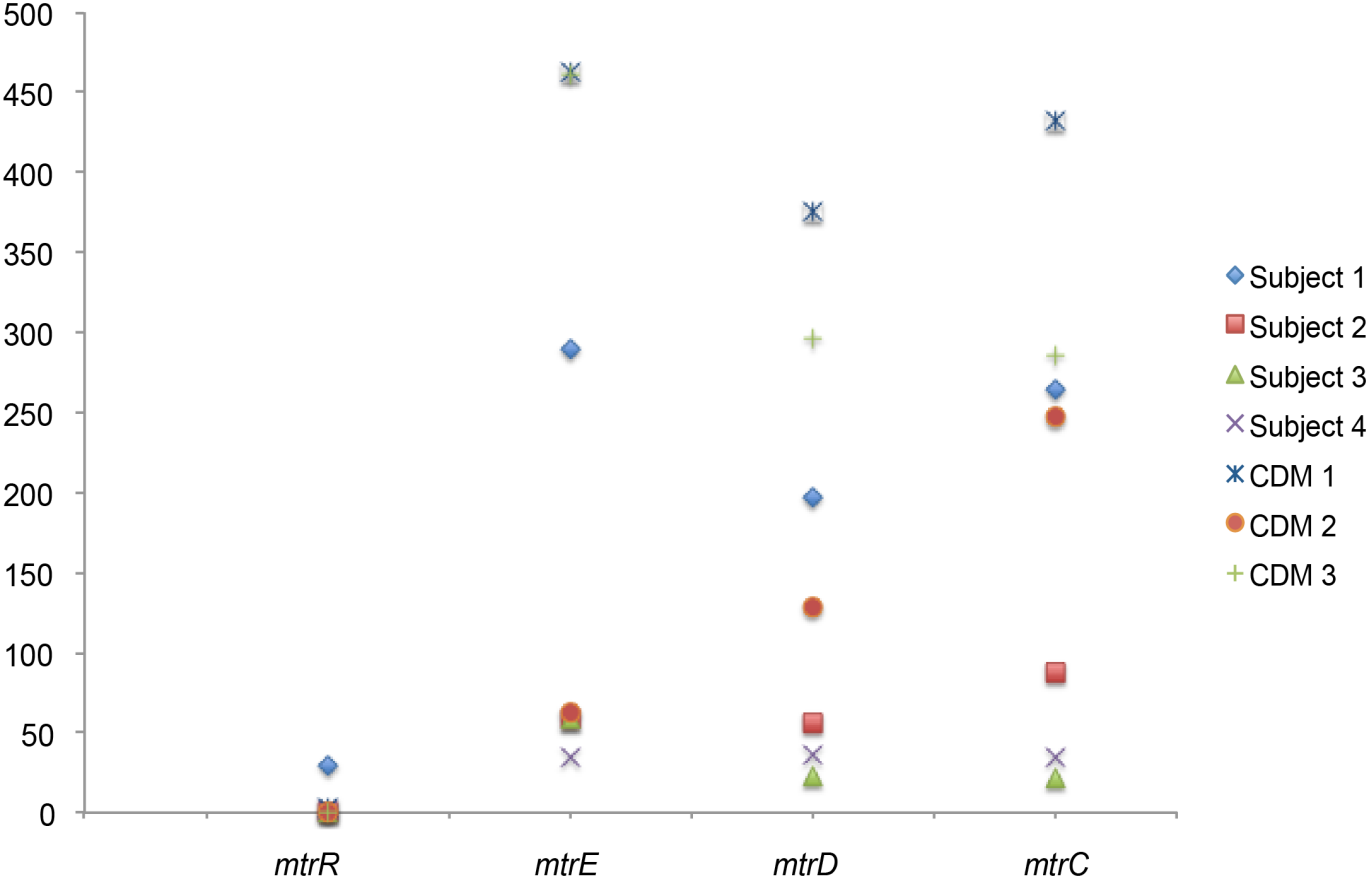
Expression of the Gonococcal *mtrR* / *mtrCDE* genes. Expression levels, reads per kilobase per million reads, (RPKM), of each of the three genes in the *mtrCDE* operon and the *mtrR* gene are shown for each of the 4 cervico-vaginal lavage samples examined as well as in the corresponding strain grown in CDM for three of subjects.

*N*. *gonorrhoeae* strains isolated recently worldwide have shown increased resistance to antibiotics [2–4]. The infecting strains in our study also were resistant to several antibiotics (S2 Table). Thus, we examined expression of genes encoding the *mtrCDE* efflux pump [23–26], a system that exports toxic, host-derived compounds and hydrophobic antibiotics from gonococci. MtrR, the regulatory protein which represses transcription of *mtrCDE* genes [27], was also examined. Expression of all genes within the *mtrCDE* locus was found in all 4 cervico-vaginal lavage samples (Fig 2). We observed lower levels of expression of the regulatory *mtrR* gene as compared to the efflux pump components for all 4 infecting strains. In Subjects 2, 3 and 4, the *mtrR* gene was not detected at an RPKM level of at least 10. Expression of *mtrR* in Subject 1 was higher than in other subjects, corresponding to a higher expression of the *mtrCDE* genes. Expression of the *mtrA* gene, which activates the *mtrCDE* locus [28], was also detected in Subjects 1, 2 and 4 with RPKM values of 67, 31 and 167 respectively (S1 Table—Line 262). It should be noted that many of the genes in the *mtr* locus also showed high expression *in vitro*, and that *mtr* genes were not specifically regulated by infection but rather showed a pattern of expression *in vivo* consistent with low levels of the repressor and high levels of the efflux pump genes (Fig 2).

### Gonococcal gene expression during natural mucosal infection relative to expression during growth in CDM

The majority of studies aimed at characterizing the response of the gonococcus to different environmental stimuli have examined *N*. *gonorrhoeae* cultured *in vitro* (i.e. during incubation in media or with host cells) conditions that do not completely replicate the environment encountered in the human host during infection. Therefore, gonococcal genes that are expressed during natural infection may differ markedly from those expressed *in vitro*. Thus, we cultured gonococcal isolates from Subjects 1, 2, and 3 (Subject 4 was diagnosed by PCR only; no isolate was recovered) in a chemically defined medium (CDM) with added ferric nitrate (100 μM final concentration) and compared the resulting transcriptome to that expressed during infection in the female genital tract. We did not observe differences in the growth rates between the 3 strains in CDM plus ferric nitrate (data not shown). Expression levels for all genes detected under *in vitro* conditions are shown in S1 Table.

**Fig 3:**
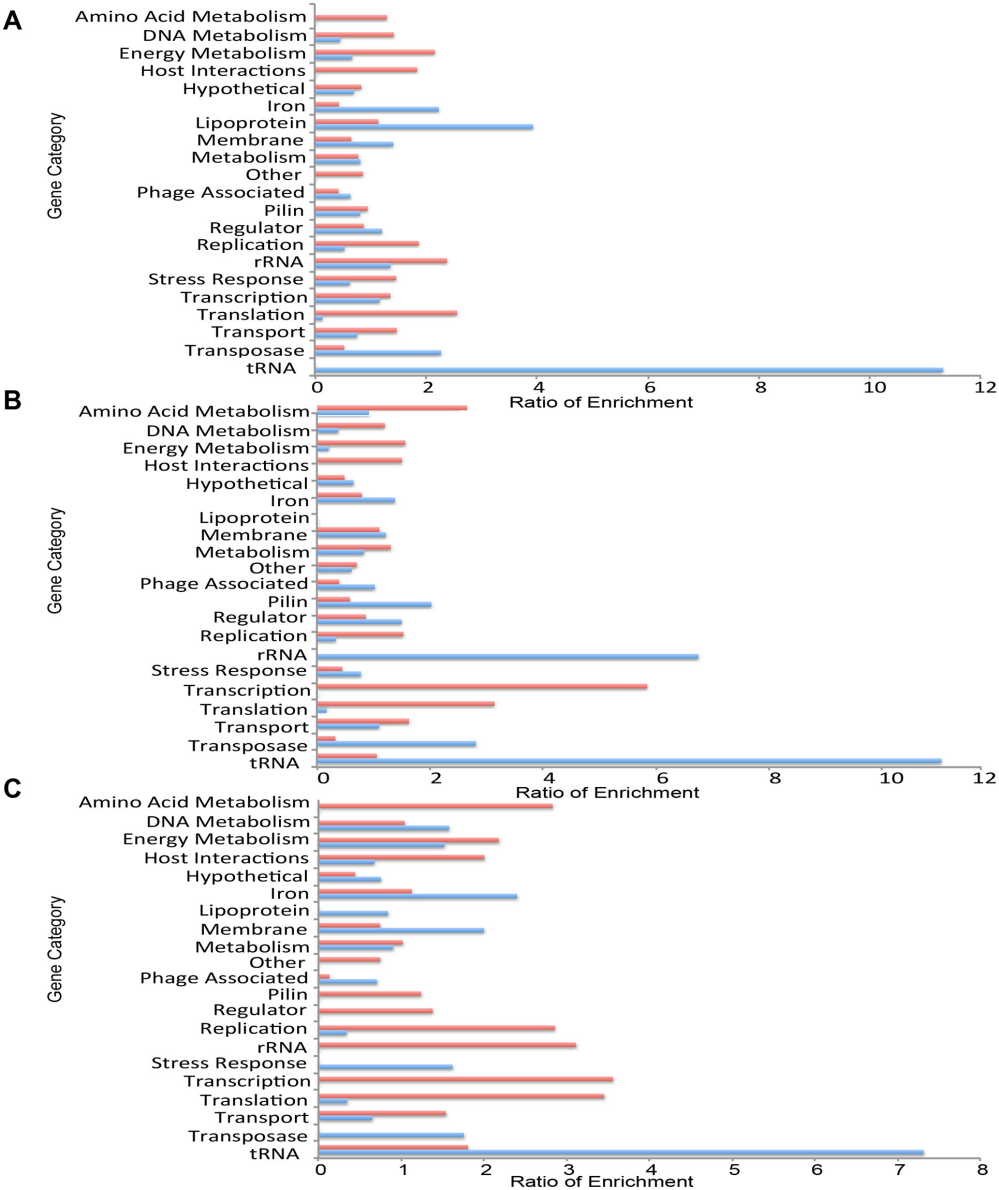
Subject-specific comparisons between transcriptomes of *N*. *gonorrhoeae* in natural infection vs. growth in CDM. *N*. *gonorrhoeae* transcriptomes observed for each subject compared with transcriptomes of corresponding infecting strains grown in CDM. Genes that showed at least a 3-fold change in expression were categorized based on gene function. **(A)** Functional enrichment of genes showing at least a 3-fold change in expression during infection of Subject 1 compared to growth of the infecting strain in CDM. Gene categories are shown in the y-axis and ratio of enrichment is shown on the x-axis. Blue bars indicate functional enrichment of genes showing increased expression during natural infection *in vivo* compared to growth in CDM. Red bars indicate functional enrichment of genes showing decreased expression during natural infection *in vivo* compared to growth in CDM. **(B)** Identical analysis for Subject 2 **(C)** Identical analysis for Subject 3.

Regulated genes were those that showed a difference in expression of at least 3-fold (up or down) in natural infection compared to growth in CDM. On average, 397 gonococcal genes, representing 20% of the genome, were regulated in each subject. Functional enrichment analysis, performed on genes showing differences in expression during natural infection compared to growth in CDM, showed enrichment of genes encoding for tRNA, iron, membrane, transposase and lipoproteins among genes whose expression was increased *in vivo* compared to growth in CDM. In contrast, metabolism, growth and housekeeping functions were enriched in genes showing decreased expression *in vivo* compared to CDM (Fig 3). Subject-specific gene expression patterns appeared overall remarkably similar, although subtle differences were also noted between subjects. For example, in the comparison between *in vivo* gene expression and growth in CDM, pilin-related genes were enriched among genes showing increased expression in Subject 2, enriched among genes showing decreased expression in Subject 3 and approximately equal Subject 1. A similar variability was observed for stress response genes, where enrichment among genes with increased expression was observed in Subject 3 but not in Subject 1 or 2. Finally, genes encoding lipoproteins were enriched among genes with increased expression in Subject 1 but not in Subjects 2 or 3. Overall, we observed general trends and a core set of genes differentially expressed in all subjects regardless of the specifics of infection. However, the subtle differences in regulation and expression of gonococcal genes, for example those involved in pilin and stress response, indicate that the genital tract of each subject was unique.

### Regulation of *N*. *gonorrhoeae* genes encoding DNA binding proteins during natural mucosal infection relative to growth in CDM

Regulatory proteins that bind DNA appear to play a prominent role in *N*. *gonorrhoeae* infection of the genital tract in women. Among the 3 subjects, each examined individually, 14 DNA-binding regulators were differentially expressed during natural infection as compared to growth in CDM, with increased expression of 6 and decreased expression of 8 regulatory protein genes. Certain genes that encode DNA binding proteins were regulated in multiple subjects, for example an AraC-like regulator (NGK_2588) was increased in Subjects 1 and 2, a two-component regulator (NGK_2403) was decreased in Subjects 2 and 3, and a CysB-like regulator decreased (NGK_1878) in Subjects 1 and 2 (Table 3). The *fur* gene also showed lower levels in RNA isolated from Subjects 2 and 3 compared to growth in CDM.

**Table 3:** Gonococcal regulators differentially expressed *in vivo* versus *in vitro*.

Locus Tag	Gene	Subject # or Composite View**
**Increased Expression** *		
NGK_0127	transcriptional regulator NrdR	1
NGK_0157	protein BasS	2
NGK_1125	NarL/NarP	2, Comp.
NGK_1256	Regulator of cell morphogenesis and NO signaling	2
NGK_1277	Putative HTH-type transcriptional regulator NMB1378	1
NGK_1600	MtrR protein	1
NGK_2588	AraC family transcriptional regulator	1, 2, Comp.
**Decreased Expression** *		
NGK_0289	GntR family transcriptional regulator	1
NGK_0454	integration host factor subunit alpha	1, 2, 3
NGK_0729	recombination regulator RecX	1
NGK_0935	putative ATP-binding protein	2
NGK_1099	protein DbhA	2, 3
NGK_1653	Regulatory protein NosR	1
NGK_1662	transcriptional regulator, AsnC family	3
NGK_1685	putative transcriptional regulator, repressor	3
NGK_1878	transcriptional regulator CysB-like protein	1, 2
NGK_2333	BolA/YrbA family protein	3
NGK_2403	Putative two-component response regulator	2, 3
NGK_2458	OxyR	1, 3
NGK_2495	Fur family ferric uptake regulator	2, 3

* Gonococcal regulators that showed increased or decreased expression (> 3-fold) *in vivo*, compared to expression of corresponding *N*. *gonorrhoeae* grown *in vitro* in CDM + ferric nitrate

^#^ Transcriptome of *N*. *gonorrhoeae* in the cervico-vaginal lavage (CVL) samples from Subjects 1, 2 or 3 compared to the transcriptome of the corresponding strain of *N*. *gonorrhoeae* isolated from Subjects 1, 2 or 3 (no strain was isolated from subject 4)

** Composite view: Expression of designated gene from CVLs from any of the 4 subjects.

### Composite analysis of gonococcal gene expression during natural mucosal infection relative to expression during growth in CDM

**Fig 4:**
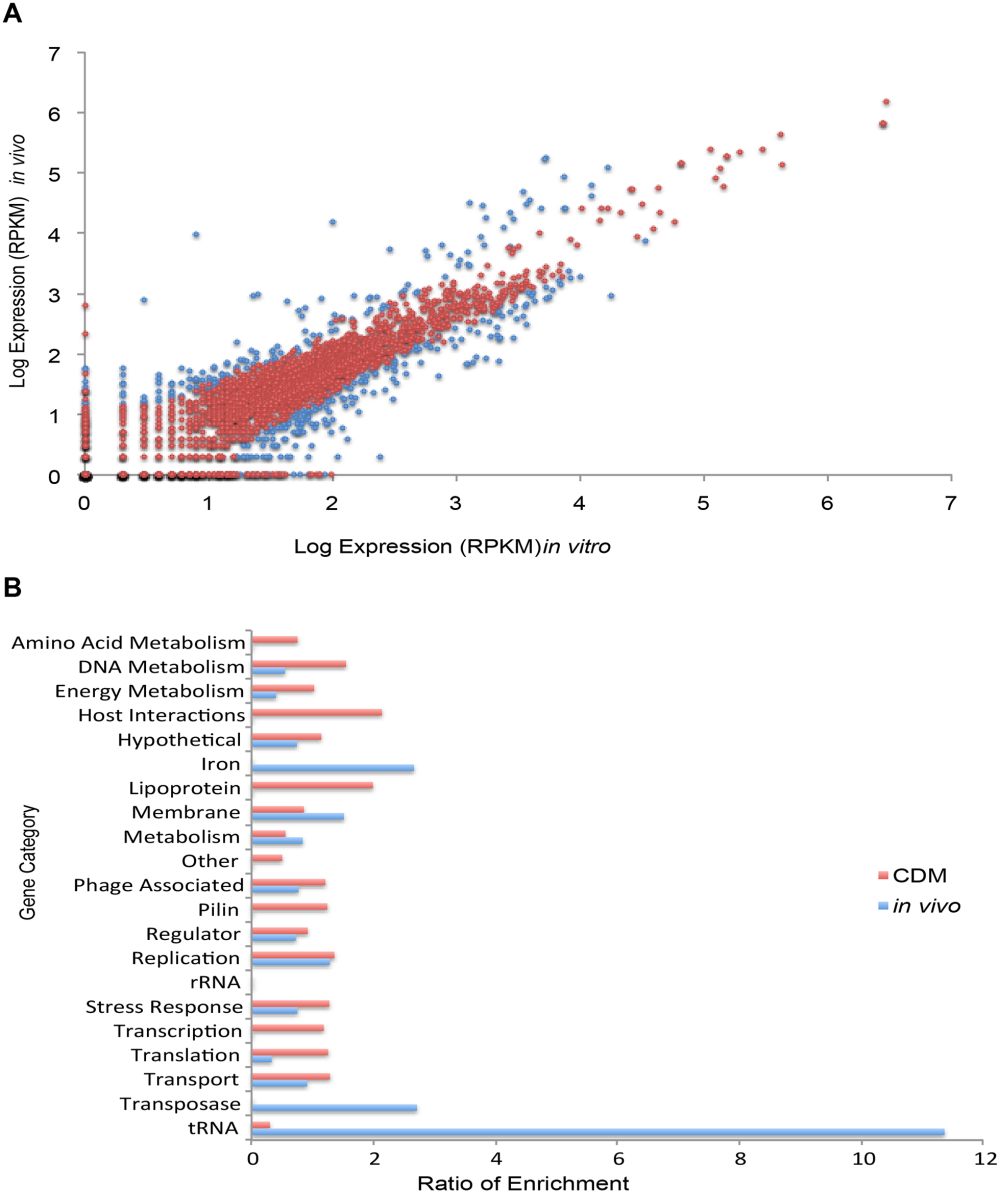
Composite comparisons between transcriptomes of *N*. *gonorrhoeae* in natural infection vs. growth in CDM. **(A)** Log values of expression (RPKM) levels are indicated. Expression levels in all 4 cervico-lavage samples were averaged and compared to the average expression levels of the three isolated *N*. *gonorrhoeae* strains grown *in vitro* in CDM supplemented with ferric nitrate (100 μM final concentration). Genes in blue had q-values < 0.05. **(B)** Functional enrichment of genes showing changes in expression when examining gonococcal transcriptomes during natural infection compared to growth in CDM supplemented with ferric nitrate (100 μM final concentration). Availability of several subject samples permitted statistical analysis and genes were considered to be differentially expressed between two samples if the q value was < 0.05 and the fold change > 2. Blue bars indicate functional enrichment of genes showing increased expression during natural infection *in vivo* compared to growth in CDM. Red bars indicate functional enrichment of genes showing decreased expression during natural infection *in vivo* compared to growth in CDM.

Several gonococcal genes were regulated in a similar fashion in each subject, suggesting that a common response of the gonococcus may be dependent on intrinsic features of the human female genital tract. Thus, we next examined the combined results from the 4 cervico-lavage samples to define an ‘average’ expression of each gonococcal gene during infection (S3 Table). As a comparison, we also combined the data resulting from the *in vitro* growth of the 3 available infecting strains, and lastly, used these averages to compare *in vivo* and *in vitro* gene expression. We identified 305 genes with statistically significant changes in expression, defined as a q-value of <0.05 and > than a 2-fold change in expression. A total of 140 genes showed increased expression and 165 decreased expression during *in vivo* vs. *in vitro* growth (S4 Table; and Fig 4A). Functional enrichment analysis was performed to define how the gonococcus alters gene expression during infection (Fig 4B and 4C). We observed that genes involved in membrane composition/function, iron scavenging, transposases and tRNAs were strongly enriched among genes with increased expression *in vivo* compared to growth in CDM. In contrast, several housekeeping genes were enriched among genes showing decreased expression, suggesting changes in growth and replication rates of *N*. *gonorrhoeae* during infection compared to growth in CDM (Fig 4B and 4C). Other categories such as pilin and lipoprotein genes also were enriched among genes showing decreased expression despite the fact that when examining subjects individually these genes were sometimes found to be enriched among genes showing increased expression. Interestingly, 20 genes encoding phage-associated proteins showed statistically significant regulation, with seven of these genes showing increased expression during infection as compared to growth in CDM (S4 Table). Phage-associated genes were also slightly enriched among genes showing decreased expression *in vivo*. The expression levels of all genes from cervico-vaginal lavage samples compared to strains grown in CDM are shown in S3 Table.

A small number of genes that encoded DNA binding regulatory proteins also exhibited statistically significant regulation when infection *in vivo* vs. growth *in vitro* in CDM was compared. Some were the same regulatory proteins that showed changes in expression when individual subjects were examined; for example an ArsC transcriptional regulator (NGK_2588) was expressed approximately 4-fold higher during *in vivo* infection vs. growth *in vitro* and was also regulated in Subjects 1 and 2 individually (Table 3).

We also identified 75 non-coding RNAs between 30–250 nucleotides in length in the composite analysis that possibly represented sRNAs (S3 Table). Expression of putative sRNAs was observed both as antisense transcripts opposite known ORFs and from intergenic regions. Ten of these sRNAs showed increased expression during natural infection compared to growth in CDM; 16 sRNAs showed decreased expression (S4 Table). Several of the sRNAs expressed during infection have previously been identified in *N*. *gonorrhoeae* under a variety of *in vitro* conditions [8,29]. For example, the sRNA NrrF exhibited statistically significant increased expression *in vivo* as compared to *in vitro*. We also detected transcription of the sRNAs 4.5S and M1 [30]. Other sRNAs, including a total of ~50% of sRNAs that had been experimentally verified in a previous study [8] were also detected, including one at genomic coordinates 1201200–1201287,which showed decreased expression *in vivo* compared to *in vitro* (S4 Table) [8]. We did not detect expression of the pilin antigenic variation small RNA [5]. A large number of transfer RNAs (tRNAs) also exhibited statistically significant changes in expression (S4 Table). Most tRNAs were detected at higher levels during natural infection compared to growth in CDM. Of 33 tRNAs that showed significant regulation, only one was expressed at higher levels during growth in CDM compared to natural infection.

### Expression and regulation of gonococcal genes that encode for iron scavenging proteins during natural mucosal infection relative to growth in CDM

**Fig 5:**
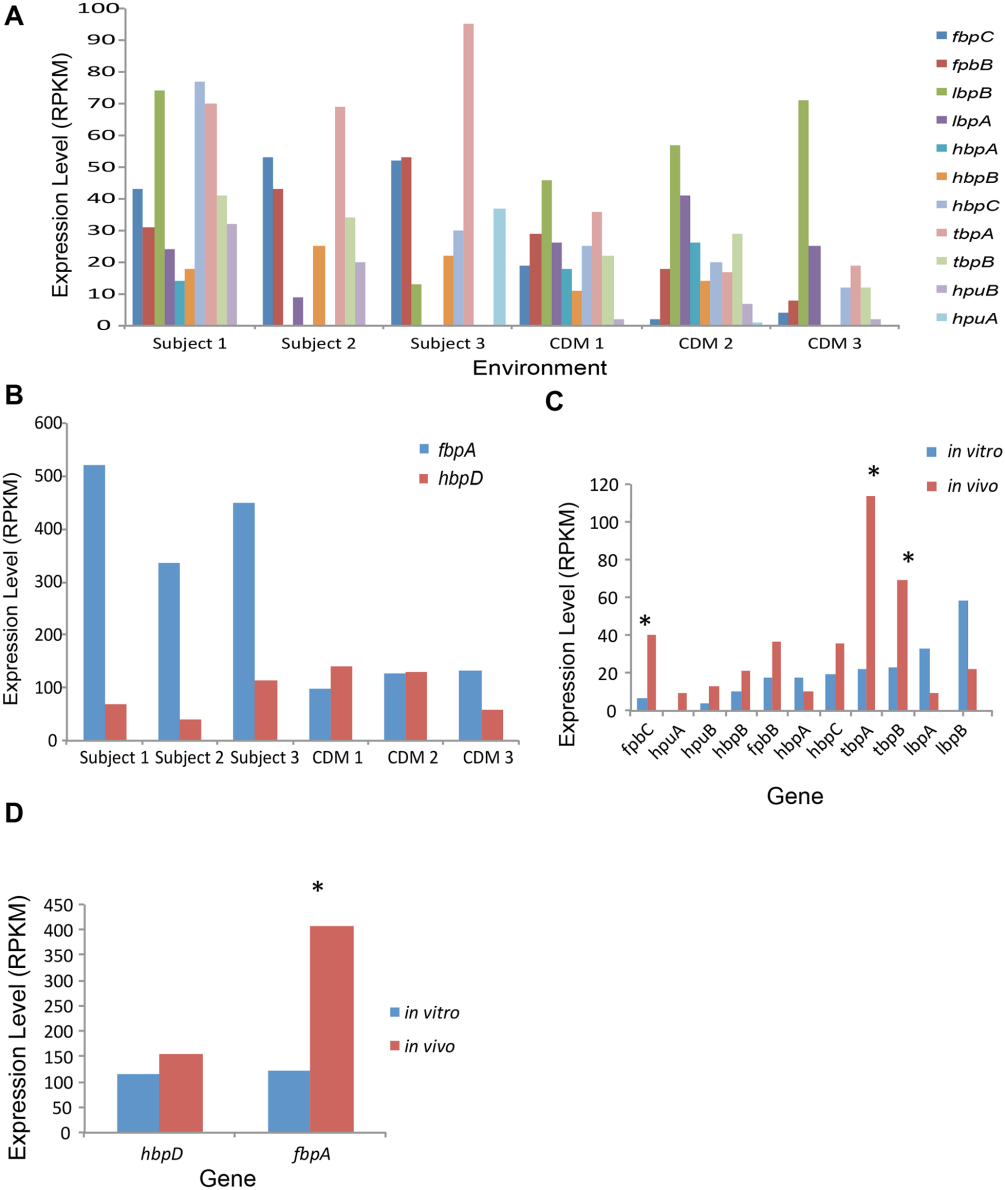
Expression of Gonococcal *fbp* and *tbp* genes. **(A)** Expression levels of *fbpC*, (blue) *fbpB* (red), *lbpB* (green), *lbpA* (purple), *hbpA* (cyan), *hbpB* (orange), *hbpC* (light blue), *tbpA* (pink), *tbpB* (light green), *hpuB* (light purple), and *hpuA* (teal) genes in RNA isolated from individual cervico-lavage samples compared to RNA isolated from the corresponding infecting *N*. *gonorrhoeae* strains grown *in vitro* in CDM supplemented with ferric nitrate (100 μM final concentration). **(B)** Expression levels of *fbpA* (blue) and *hpbD* (red) genes in RNA isolated from individual cervico-lavage samples compared to RNA isolated from the corresponding infecting *N*. *gonorrhoeae* strains grown *in vitro* in CDM supplemented with ferric nitrate (100 μM final concentration). These genes were expressed at significantly higher levels and were placed in a separate graph for ease of viewing. **(C)** Average expression levels of *fbpC*, *fbpB*, *lbpB*, *lbpA*, *hbpA*, *hbpB*, *hbpC*, *tbpA*, *tbpB*, *hpuB*, and *hpuA* gene expression in RNA isolated from 4 cervico-lavage samples (**red bars**) and average expression levels from the 3 isolated *N*. *gonorrhoeae* strains grown *in vitro* in CDM supplemented with ferric nitrate (100 μM final concentration) (**blue bars**). Expression levels (RPKM) are shown on the x-axis and represent averages of all cervico-vaginal lavage samples or all isolated strains grown in CDM. * Indicates a q-value of < 0.0001. **(D)** Average expression levels of *fbpA* and *hpbD* gene expression combined in RNA isolated from 4 cervico-lavage samples (**red bars**) and average expression levels from the 3 isolated *N*. *gonorrhoeae* strains grown *in vitro* in CDM supplemented with ferric nitrate (100 μM final concentration) (**blue bars**). Expression levels (RPKM) are shown on the x-axis and represent averages of all cervico-vaginal lavage samples or all isolated strains grown in CDM. * Indicates a q-value of < 0.0001.

Previously, we reported that the gonococcal iron and Fur-regulated genes were expressed in urethral and cervico-vaginal lavage samples obtained from men and women with gonococcal infections [16,17]. While these studies confirmed expression of *tbp* and *fbp* genes during infection, they did not address differential expression relative to corresponding infecting strains grown *in vitro*. In the current study we examined expression of *tbp* and *fbp* genes in the three culture-positive subject samples and compared this expression to the corresponding strains grown *in vitro*. Predominantly, increased expression of iron regulated *tbpA* and *tbpB* genes was seen in samples from each subject as compared to expression *in vitro*, although expression in individual samples varied. We also observed increased expression of *fbpABC* genes *in vivo* as compared to expression *in vitro* (Fig 5). Analysis of average expression of *tbpAB* and *fbpAC* genes from all cervico-vaginal lavage samples also revealed statistically significant increased levels compared to organisms growth in CDM with ferric nitrate (q < 0.0001; Fig 5). Consistent with these results, the repressor of these genes, Fur, was expressed at lower levels during natural infection compared to growth in CDM with ferric nitrate (Table 3 and S3 Table). Other iron-regulated genes also showed regulation; both *hpuA* and *hpuB* were highly expressed *in vivo* as compared to *in vitro*, and two out of four heme utilization proteins also showed a similar pattern (although not statistically significant). In contrast, lactoferrin binding proteins showed higher expression *in vitro* compared to *in vivo* (Fig 5). Because *in vitro* growth was performed under iron-replete conditions (when iron scavenging genes exhibit low expression), the high *in vivo* expression of *tbp*, *fbp*, and *hpu* genes, as well as NrrF, combined with the low expression of Fur suggests that gonococci encounter an iron-deplete environment in the female genital tract.

## Discussion

In this study we report the complete gonococcal transcriptome expressed during infection in women. Our results show that more than 65% of the genome was transcribed and detected. Genes that were highly expressed across all subjects during infection included genes associated with stress response, outer membrane and hypothetical proteins, and a high number of phage genes which previously have not been well characterized in *N*. *gonorrhoeae*. Phage associated genes have been shown previously to be intimately involved in infection including in the closely related human pathogen *N*. *meningitidis* [31–33]. While prior studies have reported the presence of at least 9 complete phage genomes within the genome of *N*. *gonorrhoeae*, the function of these phage genomes has not been investigated [34,35]. A recent study reported on the possible function of these phage proteins in the context of infection [36]. Prior studies from our group identified a phage-associated DNA binding regulatory protein that controls genes required for gonococcal infection of human epithelial cells and colonization in a mouse model [37]. Our analysis showed that 20 phage-associated genes were regulated during infection as compared to growth *in vitro* and further analysis of these genes may provide new information on the function of these genes during human gonococcal infection.

Of particular relevance in light of an alarming increase in antibiotic-resistant strains of *N*. *gonorrhoeae* were the high levels of expression of the multiple transferable resistance (*mtr) CDE* locus genes and a corresponding low expression of the *mtrR* repressor that controls expression of the operon in the gonococcal strains and samples obtained directly from subjects. Strains isolated in this study were not resistant to ceftriaxone (the recommended treatment in the United States), but other gonococcal strains with decreased susceptibility to ceftriaxone have been isolated recently in China and in the United States [4]. The *mtrCDE* locus confers resistance to several host-derived antimicrobials, including cationic antimicrobial peptides (eg. LL-37), long chained fatty acids and other hydrophobic and artificial compounds (i.e. protegrin-1, progesterone and Triton X-100). Exposure of *N*. *gonorrhoeae* to macrolides (azithromycin), β-lactam antimicrobials (penicillin) ciprofloxacin, tetracycline, rifampicin and extended spectrum cephalosporins, results in increased MICs to these antimicrobials [23–26,38]. The *mtrCDE* locus is under the control of two classical DNA binding proteins: the Mtr repressor (MtrR) protein, which down regulates expression of the *mtrC* transcript and presumably other proteins in the operon [27], and the Mtr activator (MtrA) [28], which enhances expression of *mtrCDE*. Several studies have shown that mutations of MtrR lead to enhanced fitness of *N*. *gonorrhoeae* in a mouse model of infection, suggesting that this system may also be crucial for survival of the organism in the human host [38,39].

A parallel comparative analysis of the transcriptome of *N*. *gonorrhoeae* in individual subjects and corresponding infecting isolates grown *in vitro* in a CDM identified 140 genes with increased expression and 165 genes with decreased expression. Predictably, there were subject-specific changes in the transcriptomes of *N*. *gonorrhoeae* isolates, due to the unique genital tract environment of individual subjects, which lead to tailored and specific *N*. *gonorrhoeae* responses during infection. These specific responses are likely mediated, in part, by DNA-binding transcription factors, several of which were found to be expressed and regulated during infection, including Fur and MtrR, and an ArsR-like regulator. We have shown that the latter is crucial to *N*. *gonorrhoeae* survival during infection of female endocervical cells, and that this regulator is also regulated by Fur (C. A. Genco, unpublished data). We found that numerous other regulators showed variable expression during infection, particularly those involved in nutrient acquisition and metabolism (NGK_1662, GntR, BasS). This likely reflects the large differences in metabolite composition in the female genital tract as compared to growth in CDM. In addition to well-known transcription factors, others such as NGK_1277, NGK_1662, and NGK_1878 are relatively uncharacterized and may be involved in regulation of gene products that are crucial to gonococcal infection.

Increased expression of the iron regulated *tbpAB* and *fbpABC* genes during infection was also observed as compared to *in vitro* growth. It is important to note that our studies only examined gonococcal strains grown under iron-replete conditions *in vitro*, and that a comparison of the gonococcal transcriptome expressed during infection with other *in vitro* conditions will likely identify additional genes that are differentially expressed during infection. We have previously reported that *tbpAB* and *fbpA* are expressed *in vivo* [17]. High expression and regulation of these proteins in uncomplicated cervical infection strongly suggests that the genital tract in women is an iron-deplete environment.

In addition, a large number of non-coding RNAs, including putative sRNAs and tRNAs, exhibited differential expression during infection. Relatively little is known regarding gonococcal sRNAs and studies on these regulators in *N*. *gonorrhoeae* are scarce. Work from our group and others have described the repertoire of *N*. *gonorrhoeae* sRNAs expressed during *in vitro* growth [5–8]. It is possible that post-transcriptional regulation by sRNAs plays an important role in *N*. *gonorrhoeae* gene expression during infection because of the relatively small number of DNA binding proteins expressed by gonococci compared to other Gram-negative bacteria.

In *Streptococcus pneumoniae*, *Staphylococcus aureus*, *Enterococcus faecalis* and *Pseudomonas aeruginosa*, expression of tRNAs have been reported to play a role in the development of antibiotic resistance through changes in membrane permeability [40–42]. Thus the increased expression of tRNAs observed during natural gonococcal infection relative to growth *in vitro* may represent an infection specific response to resist host-derived antimicrobial components. The current study lacks a time course analysis of gonococcal gene expression during of natural infection; it seems Iikely that the gonococcal transcriptome changes as disease progresses from initial attachment and invasion of host cells and the recruitment of immune cells. Future studies will focus on such an analysis.

A number of bacterial factors expressed during mucosal infection likely contribute to development of gonococcal disease, to the persistent nature of the infection and to disparate symptomatic responses observed in men and women. However, the majority of these microbial factors have been identified through experimental conditions that may not faithfully replicate those encountered by bacteria in their specific host environment. It is likely that many pathogenicity-associated factors expressed during natural infection are still unknown or undetected. Our studies using RNA-seq analysis are the first to define the gonococcal global transcriptome during natural mucosal infection. Collectively, we demonstrate that a large portion of the gonococcal genome is expressed during mucosal infection and that the gonococcus responds specifically to the anti-microbial and iron-deplete nature of the lower genital tract in women. Future studies should focus on examining highly expressed genes for both therapeutic and vaccine studies, as well as genes specifically regulated as a function of infection to increase our knowledge of the molecular mechanisms of infection of this pathogen.

## Materials and Methods

### Ethics statement

All subjects provided informed written consent. The BUMC Institutional Review Boards (IRB) at the University of Massachusetts Medical School, Boston University Medical Campus (protocol number H-28858), and at the Institute of Dermatology, Chinese Academy of Medical Sciences Nanjing approved the study including the protocol described in this manuscript and have determined that the study meets the requirements set forth by the IRB.

### Subject samples

Cervico-vaginal lavage samples (for RNA isolation) and swabs (for isolation of infecting strains) were obtained from four subjects attending the National Center for Sexually Transmitted Diseases (NCSTD) in Nanjing, China. Women were the exclusive sex-contacts of male subjects who had presented to the clinic with symptomatic urethritis; men were diagnosed with gonorrhea by Gram’s stain and culture (see below). Women were screened for *N*. *gonorrhoeae* infection by culture and PCR (see below), and tested positive by one or both methods. Cervico-vaginal lavage specimens were obtained according to standard collection methods [43], also demonstrated in the Microbicide Trials Network (MTN) training video used for NIAID/NIH sponsored clinical trials. Lavage fluid was stored in RLT Buffer (Qiagen) at -80°C and shipped to the United States on dry ice. Additional specimens were used for culturing and isolation/ identification of *N*. *gonorrhoeae* as described below.

### Isolation, culture and polymerase chain reaction (PCR) to identify *N*. *gonorrhoeae* in cervical swab specimens

Cervical swab specimens were taken and inoculated onto Thayer-Martin agar plates and cultured in candle jars at 36°C for 24–48 hr. Following growth on plates, bacteria were examined by colony morphology, Gram’s stain and oxidase testing. Colonies identified as *N*. *gonorrhoeae* by these criteria were isolated and sub-cultured onto chocolate agar plates overnight. The next day, colonies were resuspended in Trypticase Soy Broth/Glycerol and frozen at -70°C. PCR testing to identify *N*. *gonorrhoeae* was performed utilizing primers directed against the gonococcal *porA* pseudogene [44] and touchdown PCR for amplification [45]. Mean inhibitory concentrations (MICs) of individual strains against: penicillin, tetracycline, spectinomycin, ceftriaxone and ciprofloxacin were determined by the agar dilution method [46].

Cervical swab specimens were also tested for *Chlamydia trachomatis* and *Mycoplasma genitalium* by PCR, and *Ureaplasma urealyticum* and *Mycoplasma hominis* by culture. Vaginal swabs were collected and tested for BV, candidiasis and trichomoniasis by wet mount microscopy. Wet mount slides were examined by trained medical technicians.

For analysis of the transcriptome of *N*. *gonorrhoeae in vitro*, individual strains were grown on gonococcal base (GCB) agar plates for 16–18 hr at 37°C in 5% CO_2_ before they were resuspended in Chemically Defined Media (CDM) [47] containing ferric nitrate (100 μM final concentration). Cultures were started at an O.D._600_ of 0.1 and grown for 3 hrs before 2 ml cultures were centrifuged at 2000 rpm for 5 min and the resulting pellet used for RNA extraction.

### RNA isolation

RNA was isolated from cervico-vaginal lavage samples or from *in vitro*-grown organisms using identical protocols to avoid method-based biases. TRIzol (Invitrogen) was used to isolate RNA directly from cervico-vaginal samples or from *in vitro*-grown *N*. *gonorrhoeae* pellets. RNA was washed twice with 70% ethanol and treated with TURBO DNase (Ambion) per the manufacturer’s instructions. Eukaryotic rRNA was depleted from samples using the RiboMinus Kit (Invitrogen). To avoid biases, *in vitro*-grown *N*. *gonorrhoeae* was also treated with the eukaryotic rRNA depletion kit. Prokaryotic rRNA was depleted using the Microbe EXPRESS Kit (Ambion). Extractions were repeated twice to ensure sufficient depletion of rRNA. At no time was EDTA used. All aqueous solutions used for RNA isolation were in Diethylpyrocarbonate (DEPC)-treated water.

### RNA sequencing and analysis

Representative samples of RNA were prepared for sequencing using BioChain’s High Yield Directional mRNA Sample Prep Kit per the manufacturer’s instructions. cDNA libraries were electrophoresed on 8% Tris/Borate/EDTA (TBE) gels for 60 min at 140 V. cDNA fragments between 200–500 base pairs were extracted from the gel in TE buffer with agitation and libraries were analyzed on an Agilent Technologies 2100 Bioanalyzer to confirm cDNA library size and quantity. Libraries were sequenced using 72 and 100 single-end base pair reads on an Illumina GAIIx or High-Seq machine respectively. All analysis of RNA-seq results was carried out using the Rockhopper program [21] with expression of genes defined in reads per kilobase per million reads (RPKM). All reads from each subject’s sample were aligned to the three fully sequenced strains of *N*. *gonorrhoeae* in NCBI (NCCP11945, TCDCNGO8107 and FA1090) and the strain with the closest alignment (NCCP11945) used for downstream analysis.

To define gonococcal genes that were regulated by infection, comparisons were made between individual subject samples that yielded an isolate (total of 3; the 4^th^ was positive by PCR only) and their corresponding infecting strains (total of 3). This analysis considered genes to be regulated if they showed a fold change of > 3 between natural infection and growth *in vitro*. Not having an isolate available, Subject 4 was not used for this analysis. An additional analysis was made between a composite average of all 4 cervico-lavage samples compared to a composite average of 3 gonococcal strains grown *in vitro*. In this second analysis, availability of more than one sample permitted statistical analysis; genes were considered to be differentially expressed between two samples if the q value was < 0.05 and the fold-change was > 2. Categorization of genes was carried out using the NCBI RefSeq public database. Gene information for the NCCP11945 strain was used, the same strain used for sequence alignment. Functional enrichment was carried out after assigning all genes in the *N*. *gonorrhoeae* genome to one of 21 different categories. Functional enrichment ratios were derived through determining the percentage of genes in a given set (for example, increased expression *in vivo* compared to growth in CDM) that were assigned to a particular function and dividing this number by the percentage of genes assigned to this function in the genome as a whole. This ratio was used to interpret how enriched a given gene set was for a particular function.

## Supporting Information

S1 Table Alignment of RNA from all Subjects and from infecting strains of Subjects 1–3 to the NCCP11945 strain of *N*. *gonorrhoeae*. * Expression is defined as the RPKM level when aligning RNA isolated from Subjects 1–4 to the NCCP11945 strain of *N*. *gonorrhoeae*. ** Expression is defined as the RPKM level when aligning RNA isolated from the infecting strain of Subjects 1–3 after 3 hours of growth in CDM to the NCCP11945 strain of *N*. *gonorrhoeae*. Subject 4 was negative for *N*. *gonorrhoeae* by plating and so an infecting strain was not isolated.
(XLSX)

S2 Table Antibiotic Resistance and Porin Serovar (Type) of infecting *N*. *gonorrhoeae* strains. * All MICs displayed as μg/mL.
(XLSX)

S3 Table Composite view of alignment of RNA from all Subjects and from infecting strains of Subjects 1–3 to the NCCP11945 strain of *N*. *gonorrhoeae*. * Expression is defined as the RPKM level when aligning RNA isolated from Subjects 1–4 to the NCCP11945 strain of *N*. *gonorrhoeae*. RNA was aligned as biological replicates of a single experiment to generate a composite alignment combining all Subjects. ** Expression is defined as the RPKM level when aligning RNA isolated from the infecting strain of Subjects 1–3 after 3 hours of growth in CDM to the NCCP11945 strain of *N*. *gonorrhoeae*. RNA was aligned as biological replicates of a single experiment to generate a composite alignment combining all *in vitro* analyses. Subject 4 was negative for *N*. *gonorrhoea*e by plating and so an infecting strain was not isolated.
(XLSX)

S4 Table Regulated Genes When Examining Composite view of alignment of RNA from all Subjects and from infecting strains of Subjects 1–3 to the NCCP11945 strain of *N*. *gonorrhoeae*. * Expression is defined as the RPKM level when aligning RNA isolated from Subjects 1–4 to the NCCP11945 strain of *N*. *gonorrhoeae*. RNA was aligned as biological replicates of a single experiment to generate a composite alignment combining all Subjects. ** Expression is defined as the RPKM level when aligning RNA isolated from the infecting strain of Subjects 1–3 after 3 hours of growth in CDM to the NCCP11945 strain of *N*. *gonorrhoeae*. RNA was aligned as biological replicates of a single experiment to generate a composite alignment combining all *in vitro* analyses. Subject 4 was negative for *N*. *gonorrhoeae* by plating and so an infecting strain was not isolated.
(XLSX)
